# Vascular endothelial growth factor gene variations as a risk predictor in disc degeneration

**DOI:** 10.1590/S1679-45082017AO4053

**Published:** 2017

**Authors:** Aline Amaro, Ana Beatriz Guerra, Matheus Pippa Defino, Luiz Angelo Vieira, Carla Peluso, Bianca Bianco, Luciano Miller Reis Rodrigues

**Affiliations:** 1 Faculdade de Medicina do ABC, Santo André, SP, Brazil.

**Keywords:** Polymorphism, genetic, Vascular endothelial growth factor A/genetics, Intervertebral disc degeneration, Polimorfismo genético, Fator A de crescimento do endotélio vascular/genética, Degeneração do disco intervertebral

## Abstract

**Objective:**

To evaluate the frequency of polymorphisms in the vascular endothelial growth factor *(VEGF)* gene, as well as to identify a potential risk haplotype among the polymorphic regions in this gene in patients with disc degeneration and in the Control Group.

**Methods:**

This study analyzed a total of 217 individuals distributed into the Disc Degeneration and Control Groups. Peripheral blood was collected from all patients to detect *VEGF* gene polymorphisms identified by qPCR (rs699947, rs1570360, rs2010963, rs833061 and rs3025039). All patients presenting disc degeneration had the confirmation by nuclear magnetic resonance test and were rated according to disc degeneration level.

**Results:**

All polymorphisms were in Hardy- Weinberg equilibrium (p>0.05) in the studied population. The genotypic frequency for Disc Degeneration and Control Group were rs699947 p = 0.475, rs1570360 p = 0.862, rs2010963 p = 0.823, rs833061 p=0.596 and rs3025039 p=0.230. In haplotype analysis, the compositions CAGGC (p=0.094) and CCGGC (p=0.054) stood out.

**Conclusion:**

The correlation between *VEGF* gene polymorphism as a risk predictor for disc degeneration was negative in the studied population. However, the *VEGF* gene has a large polymorphic region, and it is activated by various catabolic and metabolic factors in the disc degeneration process, which has not been fully elucidated.

## RESUMO

**Objetivo:**

Avaliar a frequência dos polimorfismos no gene fator de crescimento endotelial vascular *(VEGF)*, bem como identificar potencial haplótipo de risco entre as regiões polimórficas deste gene em pacientes com degeneração discal e em Grupo Controle.

**Métodos:**

Este estudo analisou 217 pacientes distribuídos nos Grupos Degeneração Discal e Grupo Controle. Foi coletado sangue periférico de todos os pacientes para a detecção dos polimorfismos do gene *VEGF* identificados por qPCR (rs699947, rs1570360, rs2010963, rs833061 e rs3025039). Todos os pacientes que apresentaram degeneração discal tiveram a confirmação por meio de ressonância magnética nuclear e avaliação do nível de degeneração do disco.

**Resultados:**

Todos os polimorfismos foram encontrados no equilíbrio de Hardy-Weinberg (p>0,05) na população estudada. A frequência genotípica para o Grupo Degeneração de Disco e do Grupo Controle foi rs699947 p=0,475, rs1570360 p=0,862, rs2010963 p=0,823, rs833061 p=0,596 e rs3025039 p=0,230. Para a análise do haplótipo, destacaram-se as composições CAGGC (p=0,094) e CCGGC (p=0,054).

**Conclusão:**

A correlação entre os polimorfismos do gene *VEGF* como preditor de risco para degeneração discal foi negativa na população estudada. No entanto, o *VEGF* possui grande região polimórfica, ativada por vários fatores catabólicos e metabólicos no processo de degeneração discal, que não está completamente elucidado.

## INTRODUCTION

The low back pain is one of the most common health problems in society - between 50 and 80% of people report an episode during the lifetime - and it is an important cause of work absenteeism and use of health services.^(^ ^1^ ^)^ Among the pathological processes, which can affect the intervertebral disc, the intervertebral disc degeneration is considered the main source factor of lower back pain and lumbosciatalgia.^(^ ^2^ ^-^ ^5^ ^)^ The degeneration occurs primarily as a result of the intervertebral disc aging, nevertheless, young patients have also been affected by disc degeneration; it is considered that genetic and environmental factors as well as risk factors like work overload, smoking and overweight^(^ ^6^ ^)^ may be influencing more and more the current population who has a poor quality of life associated with increased obesity and sedentary lifestyle combining with chronic pain.^(^ ^7^ ^)^

Anatomically, the intervertebral disc consists of three major structures: the cartilaginous end-plates, the central nucleus pulposus and annulus fibrosus located on the disk periphery.^(^ ^8^ ^,^ ^9^ ^)^ The lumbar intervertebral disk is the largest avascular tissue, and its nutrition process occurs mainly through the surrounding vasculature by diffusion from the vertebral end-plates. Therefore, blood flow changes in the intervertebral disc were suggested as causal factor in disc degeneration.^(^ ^10^ ^)^ Furthermore, it may occur a blood vessels involution on degenerated disc through the vertebral end-plate or through the annulus fibrosus.^(^ ^11^ ^)^

Once the angiogenesis occurs simultaneously with the intervertebral disc degenerative process, it is reasonable to assume that angiogenesis-related genes are candidates to determine the disc degeneration development risk.^(^ ^11^ ^)^ Thus, polymorphisms in genes previously implicated in angiogenesis may contribute to the etiology of the intervertebral disc degeneration.

One of the most important peptides related to the angiogenesis is the vascular endothelial growth factor (VEGF) coded by *VEGF* gene, which is highly polymorphic, it is located on 6p21.3 chromosome and contains 14kb in the coding region with eight exons and seven introns.^(^ ^12^ ^)^

The *VEGF* operates directly and selectively through the VEGFR-1 and VEGFR-2 receptors, expressed predominantly and perhaps exclusively in the vascular endothelium. The *VEGF* connection to these receptors causes cytoplasmic calcium influx, increasing its concentration up to four times, changes the shape, division and migration of cells. This increased venous permeability to macromolecules allows plasma proteins to spread to the extra vascular space, leading to fibrinogen coagulation and fibrin gel deposition, which acts as a provisional matrix for the new blood vessels growth. The increased microvascular permeability seems invariably precede and/or follow the angiogenesis in a range of physiological and pathological processes, which makes the *VEGF* gene to be considered an important angiogenesis mediator.^(^ ^12^ ^)^

Considering the *VEGF* influence in the new vessels formation and the importance of nutrition of the intervertebral discs for their vitality, we raised a hypothesis of a possible relation between the *VEGF* gene polymorphisms and the disc degeneration.

## OBJECTIVE

To evaluate the polymorphisms frequency and the vascular endothelial growth factor gene haplotype [-2578C/A (rs699947), −1154G/A (rs1570360), +405G/C (rs2010963), −460T/C (rs833061) and +936C/T (rs3025039)] in patients with disc degeneration and also in the Control Group.

## METHODS

We performed a cross sectional study of 217 selected patients who were divided into two groups: individuals with chronic back pain (Case Group) and healthy individuals (Control Group). They were analyzed for five polymorphisms of the *VEGF* gene [-2578C/A (rs699947), −1154G/A (rs1570360), +405G/C (rs2010963), −460T/C (rs833061) and +936C/T (rs3025039)].

Considering the Case Group, a total of 110 patients with chronic back pain were selected from the Clinical Specialties - Spinal Surgery Group, of the *Hospital Estadual Mário Covas*, coordinated by the Sector of Diseases of the Locomotor System at the *Faculdade de Medicina do ABC*, Santo André (SP) Brazil. The patients inclusion criteria were: patients with chronic low back pain (longer than three months); age under 50 years old; and magnetic resonance confirming disc degeneration in at least one disc, analyzed in the sagittal plane T2 mainly, involving the discs between L4 and L5, and L5 and S1. For the rating of the disc degeneration the Pfirrmann et al.,^(^ ^13^ ^)^ grading system was utilized, and two radiologists confirmed the disease after nuclear magnetic resonance imaging test. The exclusion criteria were: patients who underwent a previous surgical treatment; with congenital spinal deformities; and with lumbar disc herniation.

For the Control Group, samples of 107 healthy individuals with no spine deformities and no back pain history were collected; they were seen at the Outpatients Clinic of *Faculdade de Medicina do ABC*. The inclusion criteria were based on clinical observations, as follows: age between 20 and 45 years; no previous spinal surgery; no history of herniated disc treatment; no history of hospitalization due to lower back pain; no use of medication for lower back pain for longer than 7 days; and no relatives aged under 50 years with herniated disc or clinical treatment for back pain.

The recruitment of all patients and methods were performed between January 2015 and November 2016. This research was approved by the Research Ethics Committee of the *Faculdade de Medicina do ABC*, under protocol 1.142.930, CAAE: 45904515.9.0000.0082.

### Polymorphism study

A volume of 4mL of peripheral blood was collected for DNA extraction, according to the Lahiri and Nurnberger (1991) protocol, using the salting-out method. The DNA obtained was quantified and qualified by Thermo Scientific NanoDrop 2000 spectrophotometer, and diluted to a standard concentration of 25ng/uL.

The polymorphisms C-2578A/rs699947, G-1154A/ rs1570360, G+405C/rs2010963, T-460C/rs833061 and C+936T/rs3025039 (Table 1) were identified by quantitative real-time polymerase chain reaction (PCR). The TaqMan assays were commercially purchased from Life Technologies^®^ (Foster City, CA, USA). The assays were conducted with TaqMan Genotyping Master Mix with 50ng DNA per reaction. The PCR conditions were as recommended by the manufacturer: initial denaturation at 95°C (15 minutes), followed by 40 denaturation cycles at 95°C (15 seconds), and an annealing/extension at 60°C (1 minuto).

**Table 1:** Vascular endothelial growth factor *(VEGF)* gene polymorphisms and respective TaqMan assays

Gene	Localization	Polymorphism	rs	TaqMan Assay
***VEGF***	6: 43736389	C2578A	rs699947	C___8311602_10
	6: 43737830	G-1154A	rs1570360	C___1647379_10
	6: 43738350	G405C	rs2010963	C___8311614_10
	6: 43737486	T-460C	rs833061	C___1647381_10
	6: 43752536	C+936T	rs3025039	C__16198794_10

### Statistical analysis

The results were statistically analyzed using the Stata 11.0 software. Genetic Power Calculator^(^ ^14^ ^)^ was used to estimate the statistical power of the results concerning individual polymorphism data.

The χ^2^ test was used to compare the genotype and allele frequencies among the groups, and to estimate the Hardy-Weinberg equilibrium. The *odds ratio* (OR) was calculated in relation to the presence of the reference genotype using a logistic regression model. The significance level was 0.05 (α<0.05). Moreover, the association between the combined genotype of *VEGF* gene polymorphisms and the disc degeneration risk was also evaluated by the haplotype study using the Haploview software version 4.1 (http://www.hapmap.org).

## RESULTS

The sample characteristics are shown in table 2.

**Table 2:** Patient characteristics

Characteristics		Disc degeneration	Control
Age		39.2±7.58	32.3±7.17
Sex, n (%)			
	Male	53 (48)	22 (21)
	Female	57 (52)	85 (79)
Race, n (%)			
	Caucasian	62 (56)	69 (65)
	Black	32 (29)	26 (24)
	No answer	16 (15)	12 (11)

The *VEGF* gene polymorphisms analysis was performed in all patients of the study. Polymorphism and alleles frequency are observed for rs699947, rs1570360, rs2010963, rs833061 and rs3025039 in table 3. The sample size power calculated was <0.50 (0.05) to the Disc Degeneration Group considering the polymorphisms studied.

**Table 3:** Frequency of vascular endothelial growth factor *(VEGF)* gene polymorphism in the population with intervertebral disc degeneration and in the control population

*VEGF* SNP	Studied population	Genotypes			Alleles		p value	OR (95%CI)	HWE
		n (%)	n (%)	n (%)	n (%)	n (%)			
rs3025039	Control	CC	CT	TT	C	T			
		81 (73.7)	26 (23.6)	3.(2.7)	188 (85.5)	32 (15.4)	0.230	1.49 (0.83-2.65)	0.875
+936C/T	Cases	85 (79.4)	22 (20.6)	0 (0)	192 (89.7)	22 (10.3)			0.495
rs833061	Control	CC	CT	TT	C	T			
		19 (17.3)	58 (52.7)	33 (30)	96(43.6)	124 (56.4)	0.596	0.88 (0.60-1.30)	0.752
-460C/T	Cases	13 (12.2)	61 (57.0)	33 (30.8)	87 (40.7)	127 (59.3)			0.171
rs699947	Control	CC	CA	AA	C	A			
		17 (15.5)	55 (50.0)	38 (34.5)	89 (40.5)	131 (59.5)	0.475	0.76 (0.52-1.13)	0.924
-2578C/A	Cases	9 (8.4)	55 (51.4)	43 (40.2)	73 (34.1)	141 (65.9)			0.332
rs1570360	Control	GG	GA	AA	G	A			
		67 (60.9)	34 (30.9)	9 (8.2)	168 (76.4)	52 (23.6)	0.862	1.07 (0.68-1.67)	0.321
-1154G/A	Cases	64 (59.8)	38 (35.5)	5 (4.7)	166 (77.6)	48 (22.4)			0.977
rs2010963	Control	GG	GC	CC	G	C			
		49 (44.5)	51 (46.4)	10(9.1)	149 (67.7)	71 (32.3)	0.823	1.07 (0.71-1.60)	0.817
+405G/C	Cases	50 (46.8)	48(44.8)	9 (8.4)	148 (69.2)	66 (30.8)			0.867

SNP: single nucleotide polymorphism; OR: *odds ratio*; 95%CI: 95% of confidence interval; HWE: Hardy-Weinberg equilibrium.

No statistically significant differences were observed in the *VEGF* gene polymorphisms studied. Furthermore, we described the Hardy-Weinberg equilibrium, and all polymorphisms were in equilibrium in the studied population. The same data was applied to haplotype analysis to estimate whether there is a correlation among the studied polymorphisms (Table 4).

**Table 4:** Haplotype analysis of polymorphisms rs8330661, rs699947, rs1570360, rs2010963 and rs3025039 of the vascular endothelial growth factor gene in patients with disc degeneration and controls

Haplotype*	Disc degeneration (%)	Controls (%)	p value
TCGCC	26.7	26.3	0.923
TCGGC	21.7	27.9	0.136
CAAGC	19.3	19.3	0.995
CAGGC	14.7	9.5	0.094
TCGCT	5.4	4.0	0.474
CCGGC	2.4	6.1	0.054
CAAGT	3.9	3.1	0.649
CAGGT	2.5	1.7	0.584
TCGGT	2.0	1.1	0.450

* Haplotype of polymorphisms rs8330661(T/C), rs699947 (C/A), rs1570360 (G/A), rs2010963 (G/C) and rs3025039 (C/T).

Haplotyping results identified two haplotype blocks that involved all polymorphisms studied. The first, CAGGC, correlated with the presence of disc degeneration (p=0.094), whereas CCGGC was more present in the Control Group (p=0.054).

## DISCUSSION

The present study showed no correlation between *VEGF* polymorphisms and disc degeneration in Brazilians. However, we observed that the haplotype CAGGC is correlated with disc degeneration, while the haplotype CCGGC was more present in Control Group. The intervertebral disc is an avascular tissue, but the adjacent vasculature supplies adequate nutrients and removes metabolic waste, and these processes are important to maintain a healthy disk.

In the stages of angiogenesis, several factors contribute to this process: cytokines (polypeptides), enzymes, extracellular matrix components and surface molecules. Two factors primarily stimulate the growth of vascular endothelium - VEGF and FGF-2. By means of immunohistochemistry tests, David et al.,^(^ ^15^ ^)^ confirmed the presence of this VEGF polypeptide in herniated intervertebral disc and/or degenerated disc. However, how the expression in this tissue is changed in herniated or degenerated disc has not been fully elucidated.^(^ ^16^ ^)^

Some authors^(^ ^17^ ^,^ ^18^ ^)^ have associated the *VEGF* expression in degenerated disc tissue. Lee et al.,^(^ ^17^ ^)^ concluded that it would be necessary to activate interleukin (IL) 1β during the disc degeneration, so that *VEGF*, NGF and BDNF could express, resulting in the angiogenesis. However, Lu et al.,^(^ ^18^ ^)^ found a positive rate of *VEGF* expression of 73.42% (116/156) in disc degeneration. In cases the intervertebral disc tissue was normal, there was no positive expression for *VEGF*. Furthermore, this expression is greater in discs with vascular infiltration as compared to discs with no infiltration.

One possible mechanism would be polymorphisms in the *VEGF* gene, thus altering its expression. The change in the gene by polymorphisms and its expression have already been demonstrated in clinical studies. For the polymorphisms −2578A −1154A of *VEGF*, there is a decrease in *VEGF* expression, but *VEGF*-634G> C SNP was correlated with a poor capacity for the production of VEGF.^(^ ^10^ ^,^ ^19^ ^,^ ^20^ ^)^

Awata et al.,^(^ ^21^ ^)^ proposed that the *VEGF* −634C allele is associated with increased transcript levels of *VEGF*, while Lambrechts et al.,^(^ ^22^ ^)^ reported that *VEGF* −634G is associated with a lower *VEGF* expression. However, no biomarker of *VEGF* has been fully associated with back pain and its mechanisms.^(^ ^23^ ^)^

Han et al.,^(^ ^24^ ^)^ observed that the frequency of combined genotypes VEGF-2578CA + AA/-634CC is higher in patients with disc degeneration. In addition, they considered that the allele VEGF-634C can be important in susceptibility to develop degeneration. This was one of the first studies conducted in the Korean population correlating the *VEGF* gene to disc degeneration.^(^ ^25^ ^)^

In this paper, the polymorphisms rs699947, rs1570360, rs2010963, rs833061 and rs3025039 were studied and no statistically significant differences were observed in these polymorphisms in relation to disc degeneration. The frequency of polymorphisms or even between alleles did not differ between the Case and Controle Groups. However, for the haplotype analysis, we observed a trend to significance for the haplotype CCGGC, patients with this combination would show a protection for the development of disc degeneration. Studies relating to disc degeneration and *VEGF* gene polymorphisms are rare in the literature, being the present paper a highly relevant work to the academic community.

This divergence between results shows that different mechanisms can activate gene transcription of *VEGF*. Binch et al.^(^ ^16^ ^)^ studied the expression of cytokines and described the responses mediated by cytokines showing minimal effects on disc degeneration. Nonetheless, nucleus pulposus cells could potentially regulate the growth of endothelial cells in a process of disc degeneration through catabolic pathways leading to chronic lumbosciatalgia.^(^ ^26^ ^)^ It is not known exactly in which stage of disc degeneration that the neovascularization process is enabled, but some factors, such as smoking, overweight and practice of exercises, can also influence the angiogenesis process.^(^ ^15^ ^)^

Polymorphisms are important factors to understand the difference between gene expressions; for example, in a normal or pathological tissue, but today they are rarely observed in clinical practice. In this study with the Brazilian population, the *VEGF* polymorphisms were not associated with disc degeneration. However, considering that many polymorphisms may have an adding effect, the haplotype analysis can still be exploited by increasing the sample size.

The sample size in the present study is a major limitation; however, the small number of patients studied is the result of selection criteria once all patients admitted in this study presented solely disc degeneration, no history of surgery and were aged under 47 years. On the other hand, the samples were in Hardy-Weinberg equilibrium and allowed genotype and haplotype analyses. Another weakness of the study could be attributed to non-evaluation of magnetic resonance imaging tests in the Control Group, but a rigid criterion of exclusion was applied.

There are several strengths in the present study. The field of genetics has been identified as a promisor area, increasing the chance of better understanding each condition that can compromise patient health. Low back pain is often underdiagnosed, and only the symptoms are treated. Sharing new tools to diagnose, treat and prevent these conditions could be essential understand health problems.

## CONCLUSION

The presence of *VEGF* gene polymorphism as a risk predictor for disc degeneration was negative in the population studied. However, the *VEGF* gene has a large polymorphic region, and it is activated by various catabolic and metabolic factors in the disc degeneration process, which have not been fully elucidated.

## References

[1] Andersson GB (1998). Epidemiology of low back pain. Acta Orthop Scand Suppl.

[2] Luoma K, Riihimäki H, Luukonen R, Raininko R, Viikari-Juntura E, Lamminen A (2000). Low back pain in relation to lumbar disc degeneration. Spine (Phila Pa 1976).

[3] Gunzburg R, Fraser RD, Fraser GA (1990). Lumbar intervertebral disc prolapse in teenage twins. A case report and review of the literature. J Bone Joint Surgery Br.

[4] Richardson JK, Chung T, Schultz JS, Hurvitz E (1997). A familial predisposition toward lumbar disc injury. Spine (Phila Pa 1976).

[5] Simmons ED, Guntupalli M, Kowalski JM, Braun F, Seidel T (1996). Familial predisposition for degenerative disc disease: A case-control study. Spine (Phila Pa 1976).

[6] Battié MB, Videman T, Levälahti E, Gill K, Kaprio J (2008). Genetic and environmental effects on disc degeneration by phenotype and spinal level: a multivariate twin study. Spine (Phila Pa 1976).

[7] Bishwajit G, Tang S, Yaya S, Feng Z (2017). Participation in physical activity and back pain among an elderly population in South Asia. J Pain Res.

[8] Bernick S, Cailliet R (1982). Vertebral end-plate changes with aging of human vertebrae. Spine (Phila Pa 1976).

[9] Maroudas A, Stockwell RA, Nachemson A, Urban J (1975). Factors involved in the nutrition of the human lumbar intervertebral disc: cellularity and diffusion of glucose in vitro. J Anat.

[10] Niinimäki J, Korkiakoski A, Parviainen O, Haapea M, Kuisma M, Ojala RO (2009). Association of lumbar artery narrowing, degenerative changes in disc and endplate and apparent diffusion in disc on postcontrast enhancement of lumbar intervertebral disc. MAGMA.

[11] Moore RJ (2006). The vertebral endplate: disc degeneration, disc regeneration. Eur Spine J.

[12] Haro H, Kato T, Komori H, Osada M, Shinomiya K (2002). Vascular endothelial growth factor (VEGF) -induced angiogenesis in herniated disc resorption. J Orthop Res.

[13] Pfirrmann CW, Metzdorf A, Zanetti M, Hodler J, Boos N (2001). Magnetic resonance classifcation of lumbar intervertebral disc degeneration. Spine (Phila Pa 1976).

[14] Purcell S, Cherny SS, Sham PC (2003). Genetic power calculator: design of linkage and association genetic mapping studies of complex traits. Bioinformatics.

[15] David G, Ciurea AV, Iencean SM, Mohan A (2010). Angiogenesis in the degeneration of the lumbar intervertebral disc. J Med Life.

[16] Binch LA, Cole AA, Breakwell LM, Michael AR, Chiverton N, Cross AK (2014). Expression and regulation of neurotrophic and angiogenic factors during human intervertebral disc degeneration. Arthritis Res Ther.

[17] Lee JM, Song JY, Baek M, Jung HY, Kang H, Han IB (2011). Interleukin- 1β induces angiogenesis and innervation in human intervertebral disc degeneration. J Orthop Res.

[18] Lu XY, Ding XH, Zhong LJ, Xia H, Chen XD, Huang H (2013). Expression and significance of VEGF and p53 in degenerate intervertebral disc tissue. Asian Pac J Trop Med.

[19] Watson CJ, Webb NJ, Bottomley MJ, Brenchley PE (2000). Identification of polymorphisms within the vascular endothelial growth factor (VEGF) gene: correlation with variation in VEGF protein production. Cytokine.

[20] Awata T, Inoue K, Kurihara S, Ohkubo T, Watanabe M, Inukai K (2002). A common polymorphism in the 5'-untranslated region of the VEGF gene is associated with diabetic retinopathy in type 2 diabetes. Diabetes.

[21] Awata T, Kurihara S, Takata N, Neda T, Iizuka H, Ohkubo T (2005). Functional VEGF C-634G polymorphism is associated with development of diabetic macular edema and correlated with macular retinal thickness in type 2 diabetes. Biochem Biophys Res Commun.

[22] Lambrechts D, Devriendt K, Driscoll DA, Goldmuntz E, Gewillig M, Vlietinck R (2005). Low expression VEGF haplotype increases the risk for tetralogy of Fallot: a family based association study. J Med Genet.

[23] Weber KT, Satoh S, Alipui DO, Virojanapa J, Levine M, Sison C (2015). Exploratory study for identifying systemic biomarkers that correlate with pain response in patients with intervertebral disc disorders. Immunol Res.

[24] Han IB, Ropper AE, Teng YD, Shin DA, Jeon YJ, Park HM (2013). Association between VEGF and eNOS gene polymorphisms and lumbar disc degeneration in a young Korean population. Genet Mol Res.

[25] Martirosyan NL, Patel AA, Carotenuto A, Kalani MY, Belykh E, Walker CT (2016). Genetic alterations in intervertebral disc disease. Front Surg.

[26] de Campos MF, de Oliveira CP, Neff CB, Correa OM, Pinhal MA, Rodrigues LM (2016). Studies of molecular changes in intervertebral disc degeneration in animal model. Acta Ortop Bras.

